# Fosmidomycin for the Treatment of Canine Otitis Externa: A Randomised, Double‐Blinded, Controlled ‘Split Body’ Clinical Trial

**DOI:** 10.1111/vde.70049

**Published:** 2026-02-02

**Authors:** Lindsey E. Citron, Darko Stefanovski, Youwen You, Rachel Proctor, Donna Watson, Julie Randall, Christine L. Cain

**Affiliations:** ^1^ Department of Clinical Sciences and Advanced Medicine University of Pennsylvania, School of Veterinary Medicine Philadelphia Pennsylvania USA; ^2^ Department of Clinical Studies—New Bolton Center, School of Veterinary Medicine University of Pennsylvania Kennett Pennsylvania USA; ^3^ Pennsylvania Equine Toxicology & Research Laboratory West Chester Pennsylvania USA; ^4^ Matthew J. Ryan Veterinary Hospital of the University of Pennsylvania Philadelphia Pennsylvania USA

**Keywords:** antimicrobial resistance, enrofloxacin, fosmidomycin, otitis externa, *Staphylococcus*

## Abstract

**Background:**

Targeted antimicrobial therapy for canine otitis externa (OE) represents an opportunity for antimicrobial stewardship. Fosmidomycin selectively inhibits the non‐mevalonate pathway for isoprenoid biosynthesis utilised by canine‐adapted, and not human‐adapted, staphylococci.

**Objectives:**

To evaluate the safety and efficacy of fosmidomycin for treatment of bacterial OE compared with enrofloxacin.

**Animals:**

Fifteen client‐owned dogs with bilateral bacterial OE were enrolled.

**Materials and Methods:**

Fosmidomycin stability in solution for 28 days was confirmed before trial commencement. A ‘split body’ design was used: each ear canal was randomised to receive a solution of fosmidomycin or enrofloxacin applied twice daily for 28 days, combined with a tapering anti‐inflammatory course of oral prednisone. Owners and investigators were blinded to treatments. Dogs were evaluated at Day (D)0, D14 and D28 with clinical scores (0–3 otitis index score [OTIS3], ear cytological results, pain) and owner assessments (pruritus scores for each ear, quality‐of‐life scores and a hearing questionnaire). On D28, owners and investigators assessed global treatment efficacy for each ear.

**Results:**

Treatment group did not significantly influence clinical scores; cytological scores, OTIS3 scores and pruritus scores significantly improved for both groups over the trial period. Treatment efficacy for both ears was assessed as good‐to‐excellent by owners and investigators for the majority of dogs. No safety concerns were identified.

**Conclusions and Clinical Relevance:**

Fosmidomycin and enrofloxacin performed comparably for topical treatment of bacterial otitis in this study. Fosmidomycin is a promising targeted antimicrobial for canine bacterial infections while limiting selection for antimicrobial resistance in human pathogens such as *Staphylococcus aureus* .

## Abstract 2

**Background:** Targeted antimicrobial therapy for canine otitis externa (OE) represents an opportunity for antimicrobial stewardship. Fosmidomycin selectively inhibits the non‐mevalonate pathway for isoprenoid biosynthesis utilised by canine‐adapted, and not human‐adapted, staphylococci. **Objectives:** To evaluate the safety and efficacy of fosmidomycin for treatment of bacterial OE compared with enrofloxacin. **Conclusions and Clinical Relevance:** Fosmidomycin and enrofloxacin performed comparably for topical treatment of bacterial otitis in this study. Fosmidomycin is a promising targeted antimicrobial for canine bacterial infections while limiting selection for antimicrobial resistance in human pathogens such as *Staphylococcus aureus* .

## 1 Introduction

Otitis externa (OE) is a leading indication for antimicrobial prescribing in small animal practice, and an important opportunity for antimicrobial stewardship [1]. *Staphylococcus* species, particularly *S. pseudintermedius* and *S. schleiferi*—recently reclassified as coagulase‐negative *S. schleiferi* and coagulase‐positive *S. coagulans—*are the most commonly isolated bacterial pathogens in canine OE [2]. *S. aureus*, a component of the normal human skin flora as well as a culprit of skin and soft tissue infections [3], is less commonly isolated from canine ear canals [4]. An antimicrobial that targets *Staphylococcus* spp. commonly associated with canine infections while minimising acquired resistance in human‐adapted *Staphylococcus* spp. would be highly desirable for treatment of OE.

Human‐adapted staphylococci (e.g., *S. aureus*) utilise the mevalonate pathway for isoprenoid biosynthesis, compounds essential for bacterial survival [5]. By contrast, staphylococci adapted to domestic animals, such as *S. pseudintermedius*, utilise the non‐mevalonate pathway [5]. This divergent pathway presents a unique therapeutic target. Additionally, mammals lack the non‐mevalonate pathway [6].

Fosmidomycin, an antimalarial and antibiotic of the phosphonic acid class, inhibits the non‐mevalonate pathway [7, 8, 9, 10]. It was shown to be effective in vitro against staphylococci that commonly colonise companion animals and not staphylococci that more typically colonise humans [5]. Bacilli (e.g., *Pseudomonas aeruginosa* and *Escherichia coli* ) also utilise the non‐mevalonate pathway for isoprenoid biosynthesis [5, 11]. Therefore, fosmidomycin may have broader applications in mixed infections or more complex otitis cases.

This study's objective was to evaluate the safety and efficacy of fosmidomycin as a topical therapy for staphylococcal or mixed bacterial OE, and to compare its efficacy to a topical antimicrobial (enrofloxacin) that is commonly used as an otic preparation. The primary outcome of interest in this trial was antimicrobial efficacy as evidenced by significant improvement in ear cytological scores. Other investigator and owner‐assessed parameters of otitis severity and patient comfort were secondary outcome measures.

## 2 Materials and Methods

### 2.1 Ethics

The Institutional Animal Care and Use Committee of the University of Pennsylvania (protocol 806505) approved this study. All participating dog owners discussed the study with clinicians and provided written informed consent before enrollment.

### 2.2 Study Design

Client‐owned dogs with bilateral bacterial cocci‐exclusive or mixed bacterial (rods and cocci) OE, based on cytological evaluation, were enrolled in this double‐blinded, controlled study using a ‘split‐body’ design. A sample size calculation was performed with a standard deviation (SD) of 1.6 and a clinically significant detectable difference in populations (a difference in mean clinical scores of 2) from a previous clinical trial [12]. Assuming that *p* < 0.05 and 80% power, 12 ears in each group were found to be the minimum sample size.

### 2.3 Enrollment Criteria

Enrollment criteria were: age ≥ 1 year; clinical signs of bilateral ceruminous or suppurative OE; and ≥ 1+ bacterial cocci (with or without bacterial rods) and < 1+ yeast using a semiquantitative method of cytological assessment [13] over an average of 10 high power fields (oil immersion) from each ear canal.

The following withdrawal periods were enforced: 7 days for topical (otic) corticosteroids, antimicrobials or antifungals; 90 days for injectable corticosteroids; and 30 days for oral corticosteroids. Oclacitinib, ciclosporin and lokivetmab were permitted if they had been used consistently for ≥ 30 days before enrollment. Exclusion criteria were: end‐stage changes such as severe stenosis or ear canal mineralisation, or strong suspicion of concurrent otitis media (e.g., facial nerve deficits, ruptured tympanum visualised on careful otoscopic examination); inability to receive twice‐daily topical therapy at home; contraindications for use of an anti‐inflammatory tapering course of prednisone; or other unregulated systemic disease.

### 2.4 Interventions

Each ear was randomised according to a predetermined computer‐generated simple randomisation scheme to receive topical fosmidomycin or enrofloxacin solution. Fosmidomycin stability in polyethylene glycol (PEG)‐400 at room temperature (RT) for 28 days was confirmed by liquid chromatography–tandem mass spectrometry (LC–MS/MS) before trial commencement (see File S1 and Figure S1).

**FIGURE 1:**
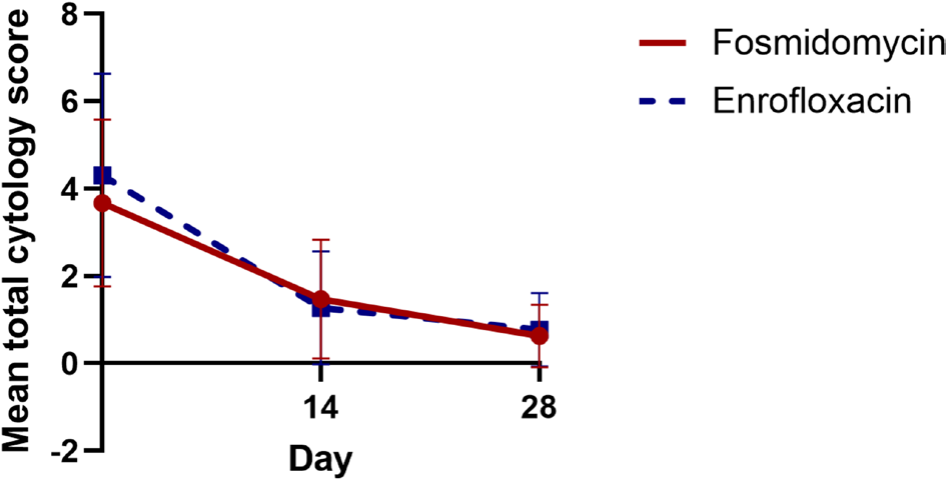
Mean total ear cytological scores at Day (D)0, D14 and D28 for ear canals randomised to receive fosmidomycin solution (circular points and solid red lines) and enrofloxacin solution (square points and dashed blue lines). Error bars indicate standard deviations at each time point.

Fosmidomycin was formulated as a 1.25 mg/mL solution: 25 mg of fosmidomycin sodium salt (Thermo Fisher Scientific) was diluted with 1.3 mL of sterile water and 18.7 mL of PEG‐400. Enrofloxacin was formulated as a 9 mg/mL solution based on published stability data [14]: 180 mg of enrofloxacin for injection (100 mg/mL; Elanco) was diluted with 18.2 mL of sterile water. Both preparations were stored at RT. Dogs weighing < 10 kg received 0.5 mL per application twice daily instilled by the owner using a 1 mL dosing syringe directed into the entrance of the external canal; dogs weighing > 10 kg received 1 mL per application twice daily using a 3 mL dosing syringe. A veterinary pharmacist formulated all otic preparations and randomised ears. Dog owners and investigators were blinded to each ear's treatment assignment. The fosmidomycin and enrofloxacin solutions were packaged in identical amber bottles and identified solely as ‘left ear medication’ and ‘right ear medication’. Separate, clearly labelled dosing syringes were provided for each ear. At‐home cleansing by owners was prohibited during the trial; cleansing with sterile saline was performed by investigators at each visit if excessive otic debris impeded otoscopic examination.

All dogs received oral prednisone at a dosage of 0.5–1 mg/kg/day for 14 days, then every other day until study conclusion (Table S1).

There were three study visits: Day (D)0, D14 (±2 days) and D28 (±2 days).

At D0, both ears were swabbed for aerobic culture and susceptibility to exclude isolation of a staphylococcal species exhibiting inherent resistance to fosmidomycin (e.g., *S. aureus*). Swabs were submitted to the Clinical Microbiology Laboratory of The University of Pennsylvania, inoculated onto blood agar (tryptic soy agar with 5% sheep's blood), MacConkey agar and colistin‐nalidixic agar (CNA) plates (Remel) and incubated for 24–48 h at 37°C. Isolates of unique morphology were subcultured for purity to appropriate media. Isolate identification and antimicrobial susceptibility testing were performed using GP and GN identification cards on the Vitek 2 automated system (bioMérieux). Calculated minimum inhibitory concentrations (MICs) were interpreted according to criteria established in Vet01S Clinical Laboratory and Standards Institute (CLSI) guidelines with breakpoints for systemic antimicrobial administration [15]. Fosmidomycin susceptibility testing was not performed as CLSI standards and clinical breakpoints have not been established for veterinary bacterial isolates.

### 2.5 Investigator Assessments

An otoscopic exam was performed at all study visits and an otitis severity score was assigned by the same investigator at each visit using the 0–3 otitis index score (OTIS3) scale [16].

Investigators evaluated otic discomfort at each study visit using a modified version of the short form Glasgow composite measure pain scale (scored from 0 to 12) [17], which was adapted to include specific signs of ear‐related discomfort (File S2).

Cytological evaluations of each ear canal, using a validated, semiquantitative (0–4+) method [13] for bacterial cocci, bacterial rods, and yeast, were performed by the same investigator at D0, D14 and D28. The average number of organisms over 10 oil immersion fields was used to assign the semiquantitative score. If only rare or occasional organisms were noted (< 1+), a score of 0.5 was assigned. The presence (1) or absence (0) of neutrophils or neutrophilic nuclear streaming also was noted and added to the score for bacteria and/or yeast to determine the total cytological score for each ear canal at each visit.

### 2.6 Owner Assessments

At each visit, owners also completed quality‐of‐life (QoL) questionnaires that assessed the effects of otitis on both dogs and their owners (QoL1/QoL2) [18, 19] and a 10 cm pruritus Visual Analogue Scale (PVAS) [20] score for each ear (0 = not itchy [normal dog]; 10 = extremely itchy).

Owners completed a questionnaire designed to assess their dog's hearing at each study visit [21]. Responses to this questionnaire indicate a hearing deficit when ≥ 2 of the eight questions indicate an abnormal hearing response. The questionnaire answers were used to formulate a numerical hearing score at each visit, with scores ≤ 5 indicative of an abnormal hearing response.

### 2.7 Global Assessment of Treatment Efficacy

At the final visit (D28), both the investigator and the owner assigned a 0–4 score (0 = no response to therapy, 1 = poor response to therapy, 2 = fair response to therapy, 3 = good response to therapy, 4 = an excellent response to therapy) for global assessment of treatment efficacy for each ear.

### 2.8 Statistical Methods

All analyses were conducted with stata 18MP (StataCorp) with two‐sided tests of hypotheses and a *p*‐value < 0.05 as the criterion for statistical significance. Descriptive analyses included median and range computation for continuous variables and tabulation of categorical variables. A test of normal distribution (Shapiro–Wilk) was performed to determine the extent of skewness of continuous variables. Frequency counts and percentages were used for summarising categorical variables.

Inference statistical analysis was based on a mixed‐effects Poisson linear regression model and the Wilcoxon signed rank test. A mixed‐effects Poisson regression model was used with treatment as the main fixed effect and visit, ear and *Staphylococcus* isolation status as confounders for outcomes: total OTIS3 score; total cytological score; individual cytological score for bacterial rods, cocci and yeast; and PVAS score. An abbreviated mixed‐effects Poisson regression model was used with a single independent variable—visit—for the outcomes pain score, hearing score and QoL score. For both Poisson regression models, random effects were set at the level of individual animals. The Wilcoxon signed rank test was used to estimate significant differences between global assessment scores by dog owners and investigators.

## 3 Results

### 3.1 Study Population

Fifteen dogs completed the trial between October 2021 and August 2023. Mixed‐breed dogs (four) and French or English bulldogs (four) were most represented, followed by pugs (two) and American cocker spaniels (two). A single dog was represented from the following breeds: English springer spaniel, beagle and West Highland white terrier. The median age was 5 years (range 1–11 years) and the median body weight was 16 kg (2.7–38.7 kg). Twelve of 15 dogs were males (11 castrated, one intact) and three of 15 dogs were females (two spayed, one intact). Dogs receive oral prednisone at a median starting dosage of 0.55 mg/kg/day (0.45–1.0 mg/kg/day), with administration decreased to every other‐day after 14 days. Full demographic information, prednisone dosages and otic solution volumes per application for each dog are included in Table S1.

### 3.2 Ear Canal Culture Results

The most commonly isolated organism on aerobic culture was *S. schleiferi*, which was classified as a single species at the time of this trial (20 ears; 19 methicillin‐susceptible and one methicillin‐resistant isolate), followed by *S. pseudintermedius* (eight ears; five methicillin‐resistant and three methicillin‐susceptible isolates). Other isolated organisms included *Proteus mirabilis* (five ears), *P. aeruginosa* (three ears) and *Corynebacterium* sp. (two ears). beta‐haemolytic *Streptococcus* sp. was isolated from one ear. Nineteen of 30 ear canals (63%) had a single organism isolated on culture while two organisms were isolated from 11 ear canals (36%). *S. aureus* was not isolated from any of the ear canals. Table 1 summarises bacterial isolates and treatment assignment for each ear canal.

**TABLE 1:** Organisms isolated from each ear canal on aerobic culture and topical treatment each ear canal was randomised to receive for 15 dogs with bilateral otitis externa.

Dog	Ear	Organism(s)	Treatment
1	Right	MSSS	F
1	Left	MSSS	E
2	Right	*Pseudomonas aeruginosa*, MSSS	E
2	Left	*P. aeruginosa* , MSSS	F
3	Right	Betahaemolytic *Streptococcus* sp.	E
3	Left	MRSP	F
4	Right	MSSS	E
4	Left	MSSS	F
5	Right	MRSP	E
5	Left	MRSP	F
6	Right	*Proteus mirabilis*, MSSS	F
6	Left	*P. mirabilis* , MSSS	E
7	Right	*P. mirabilis* , MSSS	F
7	Left	*P. mirabilis* , MSSS	E
8	Right	MSSS	F
8	Left	MSSP, MSSS	E
9	Right	MSSS	F
9	Left	MSSS	E
10	Right	MSSS	E
10	Left	MSSS	F
11	Right	*P. aeruginosa*	E
11	Left	*P. aeruginosa*	F
12	Right	MSSS*; Corynebacterium* spp.	F
12	Left	MSSS*; Corynebacterium* spp.	E
13	Right	MRSS	F
13	Left	MSSS, *P. mirabilis*	E
14	Right	MRSP	E
14	Left	MRSP	F
15	Right	*P. aeruginosa* , MSSP	F
15	Left	MSSP	E

Abbreviations: E, enrofloxacin; F, fosmidomycin; MRSP, methicillin‐resistant *Staphylococcus pseudintermedius* ; MRSS, methicillin‐susceptible *Staphylococcus schleiferi* ; MSSP, methicillin‐susceptible *Staphylococcus pseudintermedius* ; MSSS, methicillin‐susceptible *Staphylococcus schleiferi* .

### 3.3 Cytological Scores

Total cytological scores (Table 2) improved significantly over the study period for fosmidomycin and enrofloxacin‐treated ears (Figure 1). On D0, the mean cytological score across all ears was 3.9 (range 1–9). The mean total cytological score decreased to 1.4 (0–4) at D14 (*p* = 0.001) and to 0.7 (0–3) at D28 (*p* = 0.001). Treatment type did not significantly influence total cytological scores (inter‐rater reliability [IRR] 1.08; 95% confidence interval [CI]: 0.8–1.45; *p* = 0.62). At D28, 11 of 15 dogs (73%) had a total cytological score of ≤ 1 in both ears. Total cytological scores decreased by ≥ 50% for 25 of 30 ears (83%).

**TABLE 2:** 0–3 otitis severity scale (OTIS3), total ear cytological results and pruritus Visual Analogue Scale (PVAS) scores for each dog and each ear at Day (D)0 and D28.

Day 0					Day 28				
Dog	Ear	OTIS‐3	Cytology	PVAS	Dog	Ear	OTIS‐3	Cytology	PVAS
1	F	3	1.0	2.0	1	F	0	0.0	0.0
1	E	6	4.0	10.0	1	E	3	0.0	0.0
2	E	8	6.0	8.1	2	E	1	1.0	2.4
2	F	8	6.0	7.0	2	F	1	0.0	1.8
3	E	4	1.0	3.6	3	E	2	2.0	0.8
3	F	6	5.0	7.2	3	F	2	1.0	2.8
4	E	6	4.0	8.5	4	E	2	3.0	1.0
4	F	7	2.0	8.7	4	F	5	2.0	0.9
5	E	5	2.5	6.9	5	E	2	0.5	0.4
5	F	5	2.0	6.1	5	F	1	0.0	1.6
6	F	11	4.0	10.0	6	F	4	1.0	9.5
6	E	10	4.0	10.0	6	E	3	1.0	10.0
7	F	9	8.0	8.7	7	F	5	0.5	0.6
7	E	7	9.0	6.5	7	E	2	0.0	0.6
8	F	7	4.0	2.7	8	F	3	0.0	0.0
8	E	4	3.0	2.4	8	E	4	0.0	0.0
9	F	6	2.0	0.1	9	F	4	0.0	0.0
9	E	5	3.0	0.2	9	E	4	0.0	0.0
10	E	4	5.0	1.7	10	E	3	1.0	0.1
10	F	3	4.0	10.0	10	F	2	1.5	2.9
11	E	2	3.0	4.3	11	E	1	0.5	0.0
11	F	2	4.0	9.4	11	F	0	0.5	0.0
12	F	8	3.0	4.3	12	F	2	0.5	5.4
12	E	8	2.0	4.6	12	E	1	0.0	5.5
13	F	3	3.0	0.1	13	F	4	0.0	0.0
13	E	7	8.0	8.5	13	E	4	1.0	0.0
14	E	6	1.5	0.6	14	E	7	1.0	0.0
14	F	4	1.5	0.5	14	F	7	2.0	0.0
15	F	7	5.5	8.7	15	F	4	0.5	0.3
15	E	7	7.0	8.4	15	E	4	0.5	5.4

*Note:* Shaded cells represent a > 50% improvement.

Abbreviations: E, enrofloxacin; F, fosmidomycin.

Individual cytological scores for bacterial rods and bacterial cocci also improved significantly at D14 and D28. Mean cytological scores for D0, D14 and D28 (respectively): bacterial rods 1.5 (0–4), 0.5 (0–3) (*p* = 0.001) and 0.2 (0–2) (*p* = 0.001); bacterial cocci 2.1 (1–4), 0.7 (0–2) (*p* = 0.001) and 0.3 (0–2) (*p* = 0.001). Treatment type did not significantly influence cytological scores of bacterial rods (IRR 1.22; 95% CI: 0.75–1.99; *p* = 0.42) or bacterial cocci (IRR 0.89; 95% CI: 0.60–1.35; *p* = 0.59).

Mean cytological scores for yeast did not change significantly over the trial period: 0.03 (0–0.5) at D0, 0.05 (0–0.5) at D14 (*p* = 0.75) and 0.17 (0–1) at D28 (*p* = 0.14).

### 3.4 OTIS3 Scores

**FIGURE 2:**
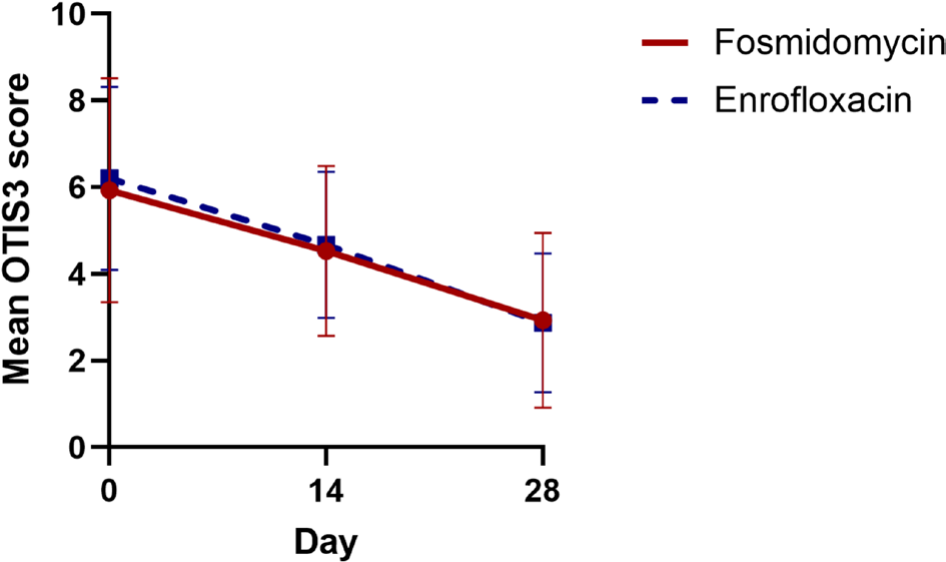
Mean 0–3 otitis severity scale (OTIS3) scores at Day (D)0, D14 and D28 for ear canals randomised to receive fosmidomycin solution (circular points and solid red lines) and enrofloxacin solution (square points and dashed blue lines). Error bars indicate standard deviations at each time point.

OTIS3 scores (Table 2) improved significantly at D14 and D28 for both fosmidomycin‐ and enrofloxacin‐treated ears (Figure 2). The mean overall OTIS3 score on D0 was 5.9 (range 2–11), which decreased to 4.6 (range 2–9) at D14 (*p* = 0.025) and to 2.9 (0–7) at D28 (*p* = 0.001). Treatment type did not significantly influence OTIS3 scores across all time points (IRR 1.01; 95% CI: 0.83–1.24; *p* = 0.87). At D28, seven of 15 (46%) of dogs had an OTIS3 score of ≤ 3 for both ears, and 12 of 15 (80%) of dogs had similar (within 1 point) or identical OTIS3 scores for both ears.

### 3.5 Pain Scores

Significant improvement in investigator‐assessed pain scores (Table S1) was noted between D0 and D28, and not at D14, with mean pain scores for dogs in both groups of 2.5 (range 0–7) at D0, 1.9 (0–6) at D14 (*p* = 0.22) and 1 (0–3) at D28 (*p* = 0.002).

### 3.6 Pruritus Scores

Significant improvement in owner‐assessed pruritus for both fosmidomycin and enrofloxacin treated ears (Table 2) was noted at D0 and D14, with a mean PVAS score of 5.7 (range 0.1–10) for all ears on D0, which improved to 1.9 (0–10) at D14 (*p* = 0.001) and 1.7 (0–10) at D28 (*p* = 0.001). Treatment group did not significantly influence PVAS scores (IRR 0.96; 95% CI: 0.75–1.23; *p* = 0.77).

### 3.7 Quality‐Of‐Life Scores

Mean QoL score for dogs (9.9 [range 1–19] at D0, 7.4 [1, 2, 3, 4, 5, 6, 7, 8, 9, 10, 11, 12, 13, 14, 15, 16, 17] at D14; *p* = 0.019) as well as the dog/owner combination (19.0 [1, 2, 3, 4, 5, 6, 7, 8, 9, 10, 11, 12, 13, 14, 15, 16, 17, 18, 19, 20, 21, 22, 23, 24, 25, 26, 27, 28, 29, 30, 31, 32, 33] at D0, 15.7 [4, 5, 6, 7, 8, 9, 10, 11, 12, 13, 14, 15, 16, 17, 18, 19, 20, 21, 22, 23, 24, 25, 26, 27, 28, 29, 30, 31, 32, 33, 34] at D14; *p* = 0.026) showed significant improvements between D0 and D14. However, there was no significant change in dog (8.1 [1, 2, 3, 4, 5, 6, 7, 8, 9, 10, 11, 12, 13, 14, 15, 16, 17, 18, 19, 20, 21, 22] at D28; *p* = 0.26), owner (9.1 [0–17] at D0, 8.2 [1, 2, 3, 4, 5, 6, 7, 8, 9, 10, 11, 12, 13, 14, 15, 16, 17, 18, 19, 20] at D28; *p* = 0.38), or combined (16.9 [4, 5, 6, 7, 8, 9, 10, 11, 12, 13, 14, 15, 16, 17, 18, 19, 20, 21, 22, 23, 24, 25, 26, 27, 28, 29, 30, 31, 32, 33, 34, 35, 36, 37, 38, 39, 40, 41, 42] at D28; *p* = 0.16) QoL scores from D0 to D28.

### 3.8 Hearing Questionnaires

The mean hearing scores (Table S1) were 5.3 (range 1–8) at D0, 4.5 (1–7) at D14, and 5.7 (1–8) at D28. Known acquired hearing loss did not occur during the study; mean hearing scores did not change significantly from D0 to D28 (*p* = 0.76). At D0, four of 15 dogs were suspected to be hearing impaired by their owners based on hearing scores (1 for Dog 6, 3 for Dog 5, and 5 for dogs 3 and 11). At D28, owner‐assigned hearing scores were 1 for Dog 6, 3 for Dog 5, 6 for Dog 3 and 7 for Dog 11.

### 3.9 Global Assessment of Treatment Efficacy

Investigators scored treatment efficacy for 12 of 15 (80%) of both ears in a set as either good or excellent (global assessment of treatment efficacy scores of 3 or 4, respectively). Owners scored treatment efficacy for 10 of 15 (66%) of both ears in a set as either good or excellent (Table 3).

**TABLE 3:** Global assessment of treatment efficacy scores for fosmidomycin (F)‐ and enrofloxacin (E)‐treated ears by investigator (clinician) and owner.

Dog	F, clinician	E, clinician	F, owner	E, owner
1	4	3	3	3
2	4	4	3	3
3	4	2	2	3
4	2	1	3	3
5	3	3	3	2
6	4	4	0	0
7	4	4	4	4
8	4	4	4	4
9	3	3	3	3
10	3	4	2	4
11	3	3	4	3
12	3	4	3	3
13	3	3	4	4
14	1	2	3	3
15	4	3	3	1

*Note:* Shaded cells indicate a pair of similar scores (within 1 point).

Abbreviations: 0, no response; 1, poor response; 2, fair response; 3, good response; 4, excellent response.

No significant differences were noted for global assessments of treatment efficacy for fosmidomycin‐ or enrofloxacin‐treated ears as scored by owners (*Z* = 0.41, *p* = 0.94) or investigators (*Z* = 0.47, *p* = 0.77). No significant differences were noted for global assessment of treatment efficacy for fosmidomycin‐treated ears as scored by investigators and owners (*Z* = 0.71, *p* = 0.55); for enrofloxacin‐treated ears as scored by investigators and owners (*Z* = 0.45, *p* = 0.72); for investigator‐assessed treatment efficacy scores for fosmidomycin‐treated ears as compared with owner‐assessed scores for enrofloxacin‐treated ears (*Z* = 0.73, *p* = 0.48); or for investigator‐assessed treatment efficacy scores for enrofloxacin‐treated ears as compared with owner‐assessed scores for fosmidomycin‐treated ears (*Z* = 0.15, *p* = 0.92).

### 3.10 Adverse Events

No adverse events attributable to study treatments were noted.

## 4 Discussion

In this study, a topical fosmidomycin solution performed comparably to topical enrofloxacin for the treatment of bacterial OE, in conjunction with a tapering course of oral prednisone. Fosmidomycin demonstrated robust in vivo antimicrobial activity against canine‐adapted staphylococci (*S. schleiferi* and *S. pseudintermedius*, including in ear canals yielding methicillin‐resistant isolates). Future studies should focus on investigating fosmidomycin as a systemic therapy for more generalised staphylococcal infections in dogs, particularly staphylococcal pyoderma that is not amenable to topical antimicrobial therapy alone.

Fosmidomycin also performed well and comparably to enrofloxacin against bacilli, including *P. aeruginosa*. Although enrofloxacin can be effective for mixed bacterial infections, widespread fluoroquinolone use may select for subsequent colonisation by methicillin‐resistant staphylococci [22, 23] or drive mutations conveying fluoroquinolone resistance in *P. aeruginosa* [24]. Enrofloxacin was chosen as the active control in this study owing to its broad spectrum activity against both Gram‐negative bacilli as well as bacterial cocci and published stability data as a sole agent in an aqueous solution [14]. Gentamicin is another broad spectrum antimicrobial commonly utilised for canine OE treatment; yet, at the time of this study's design, published stability data for gentamicin alone in solution were not yet available.

The results of this study suggest that fosmidomycin could present a promising alternative to address mixed bacterial infections, yet the impact of fosmidomycin exposure on Gram‐negative bacteria must be carefully considered. An organism's use of the non‐mevalonate pathway does not ensure susceptibility to fosmidomycin, as several different resistance mechanisms to fosmidomycin in Gram‐negative bacteria have been identified [25, 26, 27, 28, 29]. These include mutations in genes encoding for intracellular import, particularly in the glycerol‐3‐phosphate transport system [25, 26]; genes encoding for bacterial deoxyxylulose phosphate reductoisomerase (DXR) [27, 28], which is the enzyme involved in the first step of the non‐mevalonate metabolic pathway; and genes encoding for an active fosmidomycin efflux pump [25, 29]. Resistance to fosmidomycin can arise rapidly and, in *E. coli*, bacterial growth may be inhibited for only a limited period, allowing for mutant selection [25, 28]. Furthermore, mutations encoding for altered fosmidomycin transport also convey resistance to fosfomycin, which is another phosphonic acid derivative classified by the World Health Organization as a highest priority critically important antimicrobial for treatment of human infections [26, 30]. While fosmidomycin may be a judicious choice for treatment of staphylococcal infections in dogs, it should not be considered a first‐line treatment option owing to the potential to select for resistance in Gram‐negative flora.

Of the 15 dogs, seven were randomised to receive enrofloxacin solution in an ear canal from which a fluoroquinolone‐resistant (*n* = 4) or intermediate (*n* = 3) organism was isolated on culture. All of these ears had cytological improvement by D28, and all had a final total cytological score of 0–1. CLSI‐approved breakpoints for topical antimicrobial administration are lacking, and breakpoints for systemic administration are likely to underestimate antimicrobial efficacy at the high concentrations achievable with topical application to the ear canal [31]. Antimicrobial susceptibility testing of samples from the external ear canal does not reliably predict topical treatment efficacy, and results of this study support its limited utility for topical antimicrobial selection [31, 32, 33].

Otitis is an uncomfortable condition, and the process of intraotic medicating alone can temporarily increase discomfort. The investigators modified the short‐form Glasgow composite measure pain scale to identify pain‐associated behaviours specific to the ears or with ear manipulation, and scores improved over the study course. Unfortunately, there is no validated scale specific to otic pain in domestic animals, which makes interpretation of these results challenging. Regionalised pain interpretation in a nonverbal animal also is challenging, especially when anxiety in a hospital setting is common in dogs and some signs (whimpering, flinching and snapping) can be difficult to discern from discomfort [34]. Further studies should focus on the creation and validation of locoregional pain scales for domestic animals.

This study utilised an anti‐inflammatory tapering prednisone course alongside topical therapy. Glucocorticoids, topical or systemic, are standard‐of‐care as an adjunct to topical antimicrobial therapy to maximise otic patency and minimise discomfort during therapy [33, 35, 36]. Stability data for fosmidomycin combined with a corticosteroid in solution are lacking, prompting the decision to pair a tapering, anti‐inflammatory dosage of an oral corticosteroid with intraotic therapy. Topical corticosteroid monotherapy can improve PVAS and OTIS3 scores and lead to cytological resolution of mild bacterial and yeast dysbiosis [37]. The effects of systemic glucocorticoid therapy on bacterial and yeast overgrowth in the external ear are unknown; it is plausible that this too could correct acute, uncomplicated otitis. Three dogs in the present study had an initial cytological score of ≤ 3 in one ear, and one dog had cytological scores < 3 for both ears at the screening visit. For these dogs, bacteria noted in ear cytological results may have more accurately represented dysbiosis rather than true infection, and resolution may have been achieved without antimicrobial intervention. For the remaining dogs, if glucocorticoid use alone was bolstering clinical improvement, cytological scores might have rebounded when prednisone was tapered. Instead, the vast majority of OTIS3, cytological and PVAS scores remained significantly improved as prednisone was withdrawn. Because of this study's split‐body design, systemic corticosteroid administration was expected to equally impact clinical parameters such as erythema and oedema/swelling for both ear canals.

One dog (Dog 14) had an increase in OTIS3 scores for both ears over the study period, as well as < 50% cytological improvement in the enrofloxacin‐treated ear, and an increased total ear cytological score in the fosmidomycin‐treated ear (Table 2). No issues with owner compliance were identified. A flare of this dog's underlying atopic dermatitis is likely to have contributed to increased ear canal erythema, oedema and cerumen production. Resultant ear canal stenosis and ceruminous exudate may have reduced the ability of topical treatments to contact the entire ear canal surface. At‐home cleansing of the ear canals was prohibited, and this also may have impacted topical antimicrobial efficacy by reducing mechanical removal of ceruminous debris. Although a recent study of dogs with OE showed no difference in OTIS3 scores or cytological scores for bacterial cocci between ears that underwent manual cleansing and those that did not, these ears were treated with a commercially available suspension of an antibiotic, antifungal and corticosteroid [38]. The impact of ear cleansing on efficacy of an aqueous solution of a single‐agent antimicrobial for treatment of OE is unknown.

A split‐body design was selected to allow each dog to serve as its own control. Otitis externa is a dynamic process with many underlying aetiologies and complicating factors; comparing two unrelated ears, even with similar bacterial populations, would introduce more variables. One potential limitation to this design is possible systemic absorption of otic antimicrobials, leading to unintended mixing of treatment effects. Although this cannot be definitively ruled out, topical antimicrobials localised to the ear canal are unlikely to achieve substantial plasma concentrations [39].

The QoL scores were significantly improved for dogs and the dogs + owners combination at D14 yet not at D28. Successful OE treatment involves rigorous topical therapy and investigation into the primary cause of the inflammation [33, 35, 36, 40]. For this reason, owners and dogs may experience significant fatigue and inconvenience with ongoing ear management, even improved comfort and secondary infection. Owners and dogs alike experience QoL improvement when medication administration frequency is reduced with long‐acting otic preparations [41]. The burden and stress of twice‐daily topical application are suspected to be the reason for the lack of sustained improvement in QoL scores in this study.

This study identifies a therapeutic alternative that performs well against canine‐adapted staphylococci, as well as other opportunistic pathogens. Fosmidomycin therefore is a promising antimicrobial agent that minimises the selection for antimicrobial resistance in human staphylococcal pathogens such as *S. aureus*. Future studies should investigate fosmidomycin use within a larger patient subset. Prodrug inhibitors of the non‐mevalonate pathway, which do not require intracellular import like fosmidomycin and its derivatives, are also promising therapeutic options [42]. Lastly, stability studies are needed to evaluate a multi‐agent otic product containing fosmidomycin in combination with corticosteroid and antifungal agents.

## 5 Conclusion

Fosmidomycin is a well‐tolerated and effective topical antimicrobial for the treatment of canine staphylococcal or mixed bacterial OE. In this study, fosmidomycin and enrofloxacin, in conjunction with a tapering anti‐inflammatory oral corticosteroid course, demonstrated comparable improvement in ear cytological scores, as well as all other parameters of otitis severity and patient comfort. Fosmidomycin or other therapies targeting the non‐mevalonate pathway of bacterial isoprenoid biosynthesis warrant further evaluation for treatment of bacterial infections in companion animals.

## Author Contributions

**Youwen You:** investigation, writing – original draft, writing – review and editing, methodology, resources, validation, data curation, visualization. **Christine L. Cain:** conceptualization, investigation, funding acquisition, writing – original draft, methodology, visualization, writing – review and editing, project administration, data curation, supervision. **Donna Watson:** conceptualization, investigation, methodology, writing – review and editing, project administration, resources. **Lindsey E. Citron:** investigation, funding acquisition, writing – original draft, writing – review and editing, data curation, visualization. **Rachel Proctor:** investigation, writing – original draft, writing – review and editing, methodology, resources. **Darko Stefanovski:** methodology, writing – original draft, writing – review and editing, validation, formal analysis, data curation. **Julie Randall:** conceptualization, investigation, methodology, writing – review and editing, project administration, resources.

## Funding

Funding for this study was provided by awards from the PennVet Department of Clinical Sciences and Advanced Medicine Companion Animal Research Fund and the Morris Animal Foundation (D23CA‐801).

## Conflicts of Interest

The authors declare no conflicts of interest.

## Supporting information


**Figure S1:** Concentration of fosmidomycin sodium salt (in mg/mL) in polyethylene (PEG)‐400 quantified by liquid chromatography–tandem mass spectrometry over a 28 day storage time at room temperature (a) and 4°C (b). Circular points and dashed lines indicate the average concentration over time of a 5 mg/mL fosmidomycin solution at both storage conditions, while triangular points and dotted lines indicate the average concentration over time of a 1 mg/mL fosmidomycin solution at both storage conditions.

**File S1:** Methods and results for evaluation of fosmidomycin stability in solution with polyethylene (PEG)‐400 at room temperature and 4°C.

**File S2:** Adapted version of the short‐form Glasgow pain scale used by investigators to evaluate ear‐related discomfort in 15 dogs with bilateral otitis externa.

**Table S1:** Demographic data, volume of otic solutions administered per dose, initial prednisone dosages, investigator‐assessed pain scores (Day [D]0 and D28) and owner‐assessed hearing scores (D0 and D28) for 15 dogs with bilateral otitis externa. FI, female intact; FS, female spayed; MC, male castrated; MI, male intact.

## Data Availability

The data that support the findings of this study are available from the corresponding author upon reasonable request.
