# Supplementary figures and images for: Fosmidomycin for the Treatment of Canine Otitis Externa: A Randomised, Double‐Blinded, Controlled ‘Split Body’ Clinical Trial
Source: Vet Dermatol. 2026 Feb 2;37(3):427–36. doi: 10.1111/vde.70049 (PMC13167637; doi:10.1111/vde.70049)

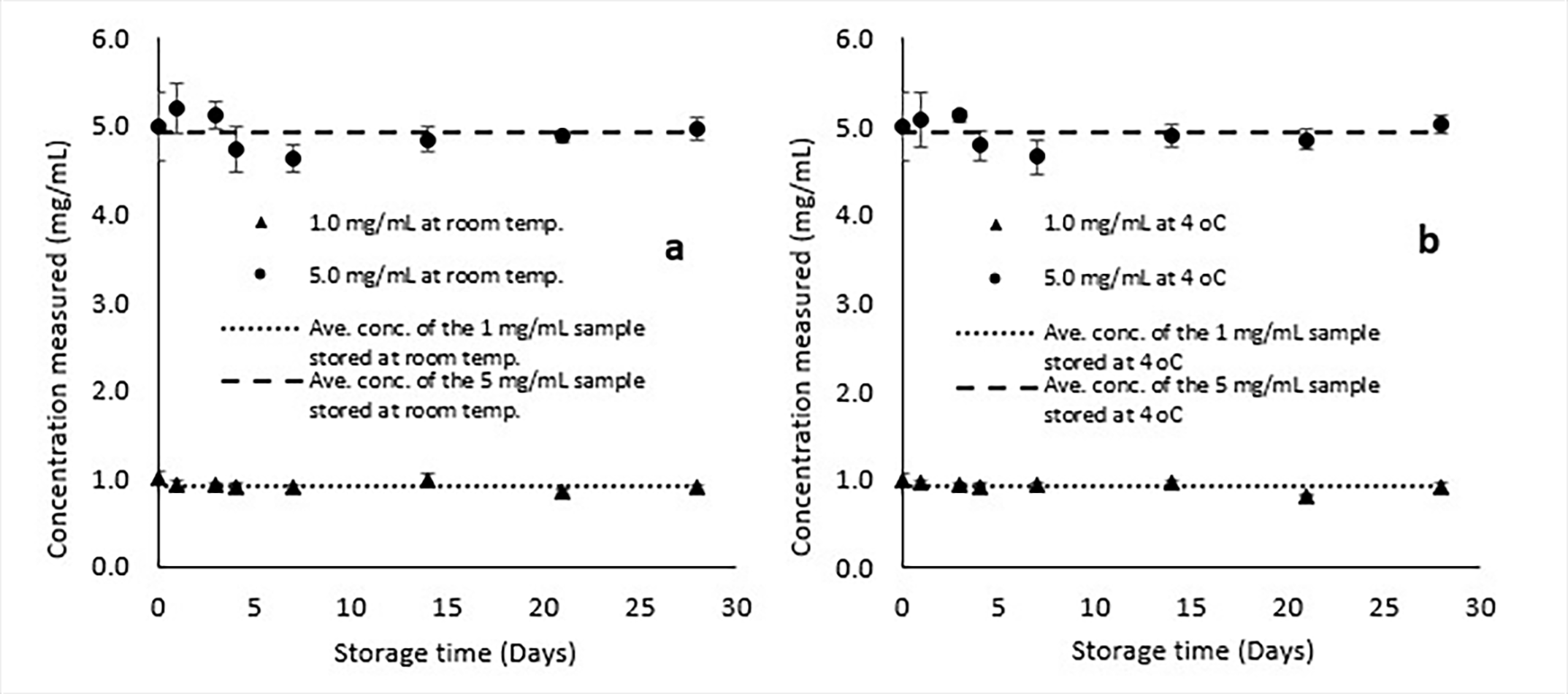

Supplement: Supplementary file 1 — Figure S1: Concentration of fosmidomycin sodium salt (in mg/mL) in polyethylene (PEG)‐400 quantified by liquid chromatography–tandem mass spectrometry over a 28 day storage time at room temperature (a) and 4°C (b). Circular points and dashed lines indicate the average concentration over time of a 5 mg/mL fosmidomycin solution at both storage conditions, while triangular points and dotted lines indicate the average concentration over time of a 1 mg/mL fosmidomycin solution at both storage conditions. [file VDE-37-427-s001.tif]
