# Supplementary material for: Fosmidomycin for the Treatment of Canine Otitis Externa: A Randomised, Double‐Blinded, Controlled ‘Split Body’ Clinical Trial
Source: Vet Dermatol. 2026 Feb 2;37(3):427–36. doi: 10.1111/vde.70049 (PMC13167637; doi:10.1111/vde.70049)
Supplement: Supplementary file 2 — File S1: Methods and results for evaluation of fosmidomycin stability in solution with polyethylene (PEG)‐400 at room temperature and 4°C. [file VDE-37-427-s004.docx]

**Fosmidomycin stability in polyethylene glycol (PEG)-400 at room temperature (RT) and 4°C**

Fosmidomycin stability in PEG-400 was evaluated at two concentrations, 1 mg/mL and 5 mg/mL, as fosmidomycin sodium salt contractions. For the 1 mg/mL solution, one bottle of fosmidomycin sodium salt (25 mg; Thermo Fisher Scientific) was dissolved in 25 mL of PEG-400. For the 5 mg/mL solution, four bottles of fosmidomycin sodium salt (25 mg each) were dissolved in 5 mL of PEG-400 individually and then combined. The prepared solutions were aliquoted into 2.0 mL glass vials, each containing approximately 1–1.25 mL. One set of aliquoted samples was immediately stored at –80°C as the Day (D)0 control sample. The remaining samples were stored under two conditions: RT (~25°C) and 4°C. Stability was assessed at the following storage times: D1, D3, D7, D14, D21 and D28. At each storage time, the stored samples were diluted in a water/acetonitrile mixture (H₂O/ACN, 60/40, v/v), and six replicates were analysed using liquid chromatography-tandem mass spectrometry (LC-MS/MS). The D0 control sample also was analysed alongside each storage time sample as a control sample. Storage time sample concentrations were normalised based on the concentration of the D0 control sample analysed on the same day.

**Fosmidomycin measurement by LC-MS/MS**

Fosmidomycin concentration was quantified using LC-MS/MS following sample dilution in a water/acetonitrile mixture (H₂O/ACN, 60/40, v/v). The analyte separation was performed on a reverse-phase ACE C18 column (7.5 cm × 2.1 mm inner diameter [i.d.], 5 μm particle size; Mac-Mod Analytical) with a guard column (1.0 cm × 2.1 mm i.d., 5 μm particle size; Mac-Mod Analytical). Fosmidomycin detection was conducted using a Sciex Qtrap 6500 mass spectrometer (AB Sciex LLC) operating in negative electrospray ionisation mode with multiple reaction monitoring (MRM). The MRM transition monitored for quantification was *m/z* 182→ *m/z* 136. The calibration range was 0.5–50 µg/mL using a quadratic regression model with 1/x weighting factor. Quality control samples at 2.5, 10 and 25 ug/mL were included in the analysis to monitor the quantification analysis accuracy. The coefficient of determination (*R*²) exceeded 0.99 for all calibration curves in this study. The samples were analysed in six replicates. Data acquisition and analysis were conducted with Analyst software (v1.6.3; AB Sciex).

**Fosmidomycin stability in PEG-400 at RT and 4°C**

Stability was assessed by monitoring changes in fosmidomycin sodium salt concentration over different storage times. Figure S1 shows that fosmidomycin sodium salt at 1 mg/mL and 5 mg/mL remained stable under both temperature conditions for >28 days. Throughout the storage period, concentrations stayed within 15% of the initial concentrations (1 and 5 mg/mL), suggesting that fosmidomycin was stable in PEG-400 solution at both RT and 4°C for 28 days.
