# Supplementary material for: Fosmidomycin for the Treatment of Canine Otitis Externa: A Randomised, Double‐Blinded, Controlled ‘Split Body’ Clinical Trial
Source: Vet Dermatol. 2026 Feb 2;37(3):427–36. doi: 10.1111/vde.70049 (PMC13167637; doi:10.1111/vde.70049)
Supplement: Supplementary file 3 — File S2: Adapted version of the short‐form Glasgow pain scale used by investigators to evaluate ear‐related discomfort in 15 dogs with bilateral otitis externa. [file VDE-37-427-s003.docx]

**Short Form Glasgow Composite Pain Scale (Adapted)**

1. **Look at the dog in the exam or treatment room**

*Is the dog?*

(i)

Quiet 0

Crying or whimpering 1

Groaning 2

Screaming 3

(ii)

Ignoring the ears 0

Holding the ears down or to the side 1

Shaking the head 2

Rubbing the ears 3

Scratching the ears 4

1. **Please palpate the ear canals and around the ear canals.**

*Does the dog?*

(iii)

Do nothing 0

Look round 1

Flinch 2

Growl or guard area 3

Snap 4

Cry 5

**Total Score (i + ii + iii) = ­­­­­**­­­­­­_________
